# A case of silicosis with *Mycobacterium avium* infection relapsed with lymphadenitis 1 year after the completion of initial treatment

**DOI:** 10.1002/rcr2.70076

**Published:** 2024-12-03

**Authors:** Taku Hatakeyama, Keiki Yokoo, Ryota Tatsuhige, Takayuki Nagao, Koki Kikuchi, Satoshi Ota, Gen Yamada, Hirofumi Chiba

**Affiliations:** ^1^ Department of Respiratory Medicine Sapporo Hokushin Hospital Sapporo Japan; ^2^ Department of Respiratory Medicine Teine Keijinkai Hospital Sapporo Japan; ^3^ Department of Infectious Diseases Teine Keijinkai Hospital Sapporo Japan; ^4^ Department of Pathology Teine Keijinkai Hospital Sapporo Japan; ^5^ Department of Respiratory Medicine and Allergology Sapporo Medical University School of Medicine Sapporo Japan

**Keywords:** lymphadenitis, *Mycobacterium avium* complex, pneumoconiosis, silicosis

## Abstract

A 71‐year‐old man with silicosis was treated for *Mycobacterium avium* infection. Antimycobacterial treatment for *M. avium* was terminated 1 year after a negative test result for the bacteria on sputum examination. One year following the treatment, the patient developed pneumonitis. In the sputum culture, growth of *M. avium* was not detected. Pneumonitis did not improve despite sufficient antibacterial therapy. Chest computed tomography scan revealed aggravated shadows of pneumonitis and swelling of supraclavicular lymph nodes. A lymph node biopsy was performed, and polymerase chain reaction testing revealed *M. avium* infection. We diagnosed the patient with pneumonitis and lymphadenitis due to recurrent *M. avium* infection. Antimycobacterial treatment with rifampicin, ethambutol, clarithromycin, and amikacin was initiated. Pneumonitis and the general status of the patient improved following the treatment. Lymphadenitis is rare in adults in the absence of any immunodeficiency condition. We speculate that silicosis is a risk factor for mycobacterial infection recurrence.

## Graphical abstract

This report describes the case of a 71‐year‐old man with silicosis with *Mycobacterium avium* infection relapsed with lymphadenitis 1 year after the completion of initial treatment. Lymphadenitis is rare in adults in the absence of any immunodeficiency condition. We speculate that silicosis is a risk factor for mycobacterial infection recurrence.

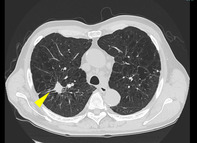

## INTRODUCTION

*Mycobacterium avium complex* (MAC) is the most frequently detected nontuberculous *mycobacterium* (NTM) species, and the incidence of MAC infection is increasing globally. Lymphadenitis caused by MAC has been recognized in many cases in children; however, the condition has rarely been reported in adults. In almost all the cases reported in adults, the individuals were immunocompromised with human immunodeficiency virus infection or received immunosuppressive therapy.^1^

Silicosis is a chronic lung disease associated with silica exposure and can be a risk factor for mycobacterial lung infections as a result of damaged pulmonary macrophages.^2^

Herein, we report the case of an adult patient with silicosis and *M. avium* lymphadenopathy who previously underwent antimycobacterial treatment.

## CASE REPORT

**FIGURE 1:**
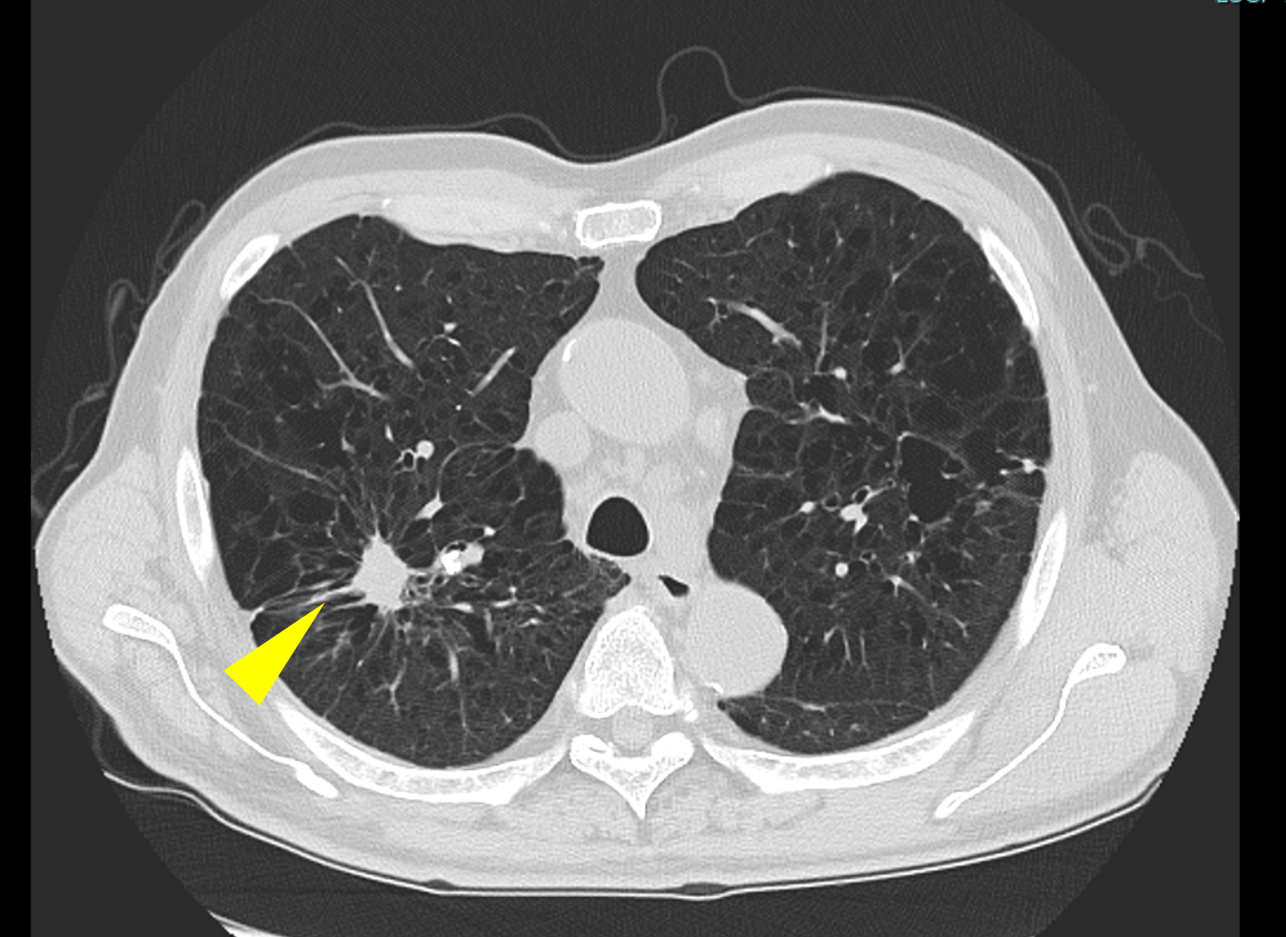
Chest computed tomography scan demonstrates a solitary silicotic nodule in the upper lobe of the right lung (yellow arrow).

A 71‐year‐old man with silicosis diagnosed on histopathology examination was treated for a non‐cavity‐type *M. avium* infection (Figure 1). He was administered rifampicin (RFP), ethambutol (EB), and clarithromycin (CAM) therapy, which was terminated 1 year after a negative result for the bacteria on sputum examination. The total duration of treatment was 1 year and 4 months. The patient was followed up with watchful observation.

**FIGURE 2:**
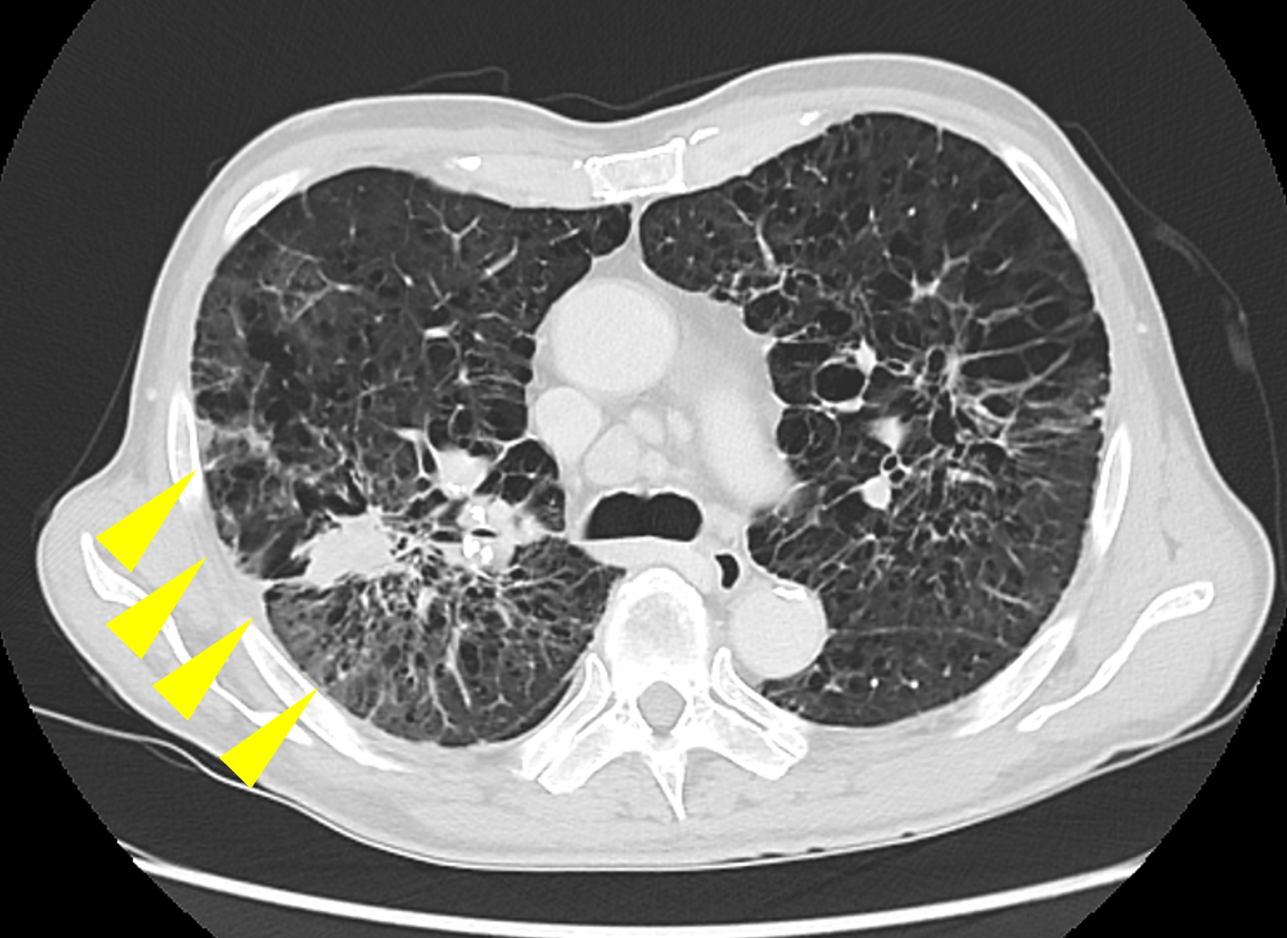
Chest computed tomography scan demonstrates a consolidation and ground‐glass opacity surrounding silicotic nodules (yellow arrow).

One year after the antimycobacterial treatment, the patient was admitted to our hospital with fever, cough, and weight loss. Consequently, he was diagnosed with pneumonia. On admission, a chest computed tomography (CT) scan revealed consolidation and ground‐glass opacity around the silicotic nodules in the right upper lobe (Figure 2). Although the sputum examination upon admission revealed *Staphylococcus epidermidis* growth, *M. avium* was not cultured.

Laboratory data demonstrated an increased white blood cell count (16,340/μL) and elevated C‐reactive protein (CRP) levels (3.03 mg/dL). Human immunodeficiency virus (HIV)‐specific antibody was negative and anti‐interferon‐γ auto‐antibody was negative. His serum immunoglobulin values were normal. Therefore, antibiotic treatment with ampicillin‐sulbactam was initiated to manage bacterial pneumonia.

However, the high fever persisted, and the white blood cell count and CRP levels gradually increased. A chest radiograph revealed an enlargement of abnormal shadows.

**FIGURE 3:**
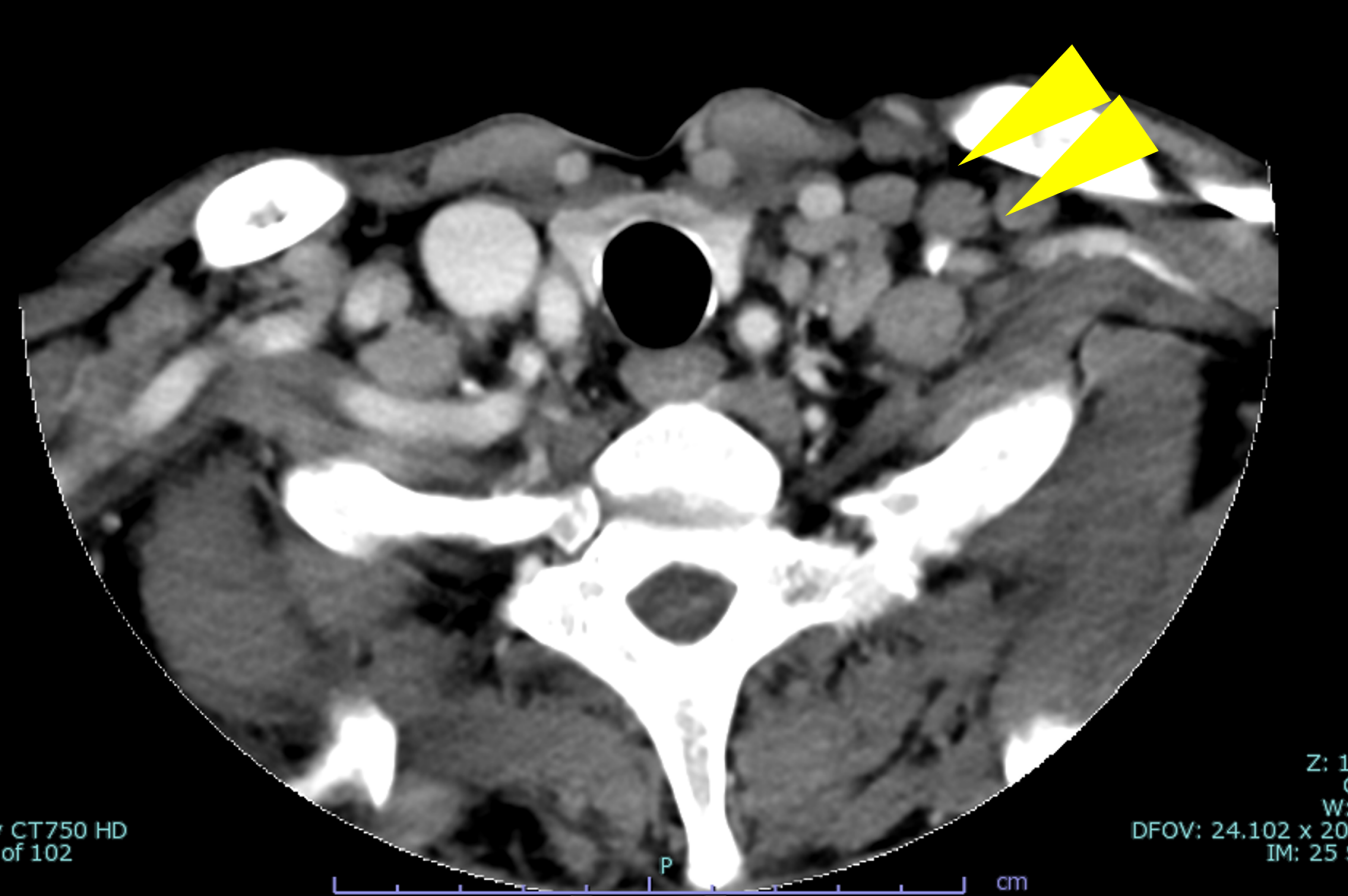
A computed tomography scan demonstrates swelling of supraclavicular lymph nodes (yellow arrow).

A contrast‐enhanced CT scan performed to investigate the cause of the fever and assess the lung shadow revealed left supraclavicular lymph node swelling (Figure 3) and enlarged consolidation around the silicotic nodules. Subsequently, a biopsy of the supraclavicular lymph node was performed. Pathology examination findings from the biopsy sample demonstrated granulomatous lesions without necrosis; however, polymerase chain reaction testing of the sample revealed *M. avium*.

Based on these results, we diagnosed the patient with lymphadenitis due to recurrent *M. avium* infection. Subsequently, treatment with RFP, EB, CAM, and amikacin (AMK) was initiated, and drug susceptibility tests were performed simultaneously. After treatment initiation, the fever subsided, and lung consolidation and laboratory findings gradually improved. As a non‐multidrug‐resistant *M. avium* strain was confirmed, AMK administration was terminated after 4 weeks, and antimycobacterial treatment with RFP, EB, and CAM was continued. No apparent recurrence of the infection was observed. The patient's general status improved, and he was transitioned to outpatient treatment.

## DISCUSSION

Clinical manifestations associated with NTM disease can be observed in various organs. The most common site of manifestation is the lungs; however, other sites, including the skin, soft tissue, and lymph nodes can also be involved. Furthermore, disseminated disease is also possibly observed.^3^

Although lymphadenitis is the most common manifestation of NTM disease in children, cases in adults are rare, except in individuals with immunodeficiency conditions, such as acquired immunodeficiency disorder and blood disorders, or those taking glucocorticoids. The predominance of MAC infection has been reported previously; however, the etiologic pathogens vary by region and country.^3^

In this case, we initially suspected a medical history of immunosuppression or an immunological disorder, however, the results of anti‐interferon‐γ auto‐antibody were negative, and no immunodeficiency condition was identified. Hence, we concluded that a history of silicosis contributed to the recurrence of *M. avium* infection. According to several reports, silicosis is a risk factor for pulmonary diseases, including NTM infection.^4^ However, the mechanism by which silicosis increases the risk of *Mycobacterium* infections remains unclear. Additionally, NTM infection is associated with functional disorders of macrophages and structural changes in the lungs due to silicosis.^2^ We speculate that if macrophages are not fully functional, the current treatment duration for NTM infection may not provide adequate control.

No standardized approach is available for the treatment of lymphadenitis due to NTM infection; however, a combination of surgical and antimycobacterial treatments is often used. A meta‐analysis of randomized and observational studies on paediatric patients with NTM lymphadenitis reported higher cure rates in patients who underwent complete surgical resection than in those who were administered antimycobacterial treatment alone.^1^

As the recurrence rate is reported to be less than 1% in adults with complete resection,^5^ surgical treatments should be selected in cases with a single lesion or minimal spread to the surrounding area.

In a few reports of NTM lymphadenitis in adults, the regimens selected for antimycobacterial therapy varied. In the present case, RFP, EB, CAM, and AMK regimen was selected for the re‐treatment of *M. avium* leading to a gradual improvement in the general status of the patient.

Although international treatment guidelines recommend that MAC infection be treated for at least 12 months after conversion to a culture‐negative state,^1^ the appropriate duration of treatment in patients with complications such as immunosuppression and silicosis remains unclear despite frequent MAC infection recurrences. In particular, no treatment recommendations exist for patients with silicosis. Further studies are needed to elucidate the epidemiology of NTM infection in patients with pneumoconiosis and to determine the appropriate duration of treatment.

## AUTHOR CONTRIBUTIONS

*Concept and design*: Taku Hatakeyama, Keiki Yokoo. *Drafting of the manuscript*: Taku Hatakeyama, Keiki Yokoo. *Critical review of the manuscript for important intellectual content*: Taku Hatakeyama, Keiki Yokoo, and Gen Yamada. *Supervision*: Keiki Yokoo.

## CONFLICT OF INTEREST STATEMENT

None declared.

## ETHICS STATEMENT

This case report was approved by the Ethics Committee (permit no. 3‐024314‐00). The authors declare that appropriate written informed consent was obtained for the publication of this manuscript and the accompanying images.

## Data Availability

The data that support the findings of this study are available on request from the corresponding author. The data are not publicly available due to privacy or ethical restrictions.
